# Variation in intraspecific demography drives localised concordance but species-wide discordance in response to past climatic change

**DOI:** 10.1186/s12862-022-01990-2

**Published:** 2022-03-22

**Authors:** Sean James Buckley, Chris J. Brauer, Peter J. Unmack, Michael P. Hammer, Luciano B. Beheregaray

**Affiliations:** 1 Molecular Ecology Laboratory, College of Science and Engineering, Flinders University, Adelaide, SA 5001 Australia; 2 Centre for Applied Water Science, Institute for Applied Ecology, University of Canberra, Canberra, ACT 2601 Australia; 3 Natural Sciences, Museum and Art Gallery of the Northern Territory, Darwin, NT 0801 Australia

**Keywords:** Coalescent analysis, Biogeography, Comparative phylogeography, Temperate Australia, Percichthyidae

## Abstract

**Background:**

Understanding how species biology may facilitate resilience to climate change remains a critical factor in detecting and protecting species at risk of extinction. Many studies have focused on the role of particular ecological traits in driving species responses, but less so on demographic history and levels of standing genetic variation. Additionally, spatial variation in the interaction of demographic and adaptive factors may further complicate prediction of species responses to environmental change. We used environmental and genomic datasets to reconstruct the phylogeographic histories of two ecologically similar and largely co-distributed freshwater fishes, the southern (*Nannoperca australis*) and Yarra (*N. obscura*) pygmy perches, to assess the degree of concordance in their responses to Plio-Pleistocene climatic changes. We described contemporary genetic diversity, phylogenetic histories, demographic histories, and historical species distributions across both species, and statistically evaluated the degree of concordance in co-occurring populations.

**Results:**

Marked differences in contemporary genetic diversity, historical distribution changes and historical migration were observed across the species, with a distinct lack of genetic diversity and historical range expansion suggested for *N. obscura*. Although several co-occurring populations within a shared climatic refugium demonstrated concordant demographic histories, idiosyncratic population size changes were found at the range edges of the more spatially restricted species. Discordant responses between species were associated with low standing genetic variation in peripheral populations. This might have hindered adaptive potential, as documented in recent demographic declines and population extinctions for the two species.

**Conclusion:**

Our results highlight both the role of spatial scale in the degree of concordance in species responses to climate change, and the importance of standing genetic variation in facilitating range shifts. Even when ecological traits are similar between species, long-term genetic diversity and historical population demography may lead to discordant responses to ongoing and future climate change.

**Supplementary Information:**

The online version contains supplementary material available at 10.1186/s12862-022-01990-2.

## Background

Understanding how or whether species may be able to adapt to current and future climatic changes is critical for conservation management of threatened taxa [1]. However, predicting the susceptibility and extent of species loss due to climate change remains a challenge. To this end, many studies have instead sought to determine ecological traits that may confer resilience or susceptibility to climate change across various taxa [2]. Ecological and physiological traits such as thermal tolerance and dispersal capacity have been shown to be critical in driving adaptation to climatic changes [3, 4]. Demographic and genetic traits such as population size, stability and standing genetic variation (SGV) are however also important in facilitating adaptation to new environmental stressors [5], and likely play a major role in species responses to climate change [6–8].

From a genetic perspective, adaptation to novel climatic conditions more often relies upon SGV than de novo mutations [9–11]. The degree to which SGV is maintained within species or populations varies substantially across taxa and is influenced by a combination of demographic, ecological and environmental factors. For example, populations occurring at the edge of a species range often have lower connectivity and genetic diversity than their more central counterparts [12], including reduced diversity in climate-associated genes [13]. In marginal populations, persistence is driven by the balance of the steepness of the selective environment and the effectiveness of selection relative to genetic drift [14]. These components may contrast with the core of the distribution, where larger carrying capacities and SGV allow populations to persist closer to their selective optimum [15]. Thus, the interaction and spatial variability of neutral (demographic) and adaptive (ecological) traits are critically important in understanding how species ranges may shift under climate change [16].

Understanding factors underlying species responses to historical climatic fluctuations provides an empirical framework for determining how species may respond to current and future environmental changes [17]. Extending phylogeographic analyses from taxon-specific studies to assessments of how species assemblages have responded to past climatic changes provides an approach to estimating the ubiquity of species responses [18]. Similar species responses (concordance) across disparate taxa often indicate that shared ecological traits underlie the response [19] or demonstrate the ubiquity in impact of the environmental change in question [20]. Contrastingly, idiosyncratic responses (discordance) are often attributed to variation in species-specific ecological traits [21]. However, intraspecific variation in demography may lead to spatial variation in the degree of concordance, even across ecologically similar species. For example, the interactive role of demography and adaptive potential may lead to intraspecific variation at local scales, even if species-wide patterns are concordant across taxa or vice versa [22, 23]. These patterns may be reflected within species range shifts over time, where intraspecific variation in demographic or ecological traits at range margins may drive interspecific discordance in species responses to environmental change.

Biogeographic regions that experienced major environmental change in the past are particularly useful for studying species responses to climate change. In this regard, the southeast Australian temperate zone provides a model region to test how species have responded to major environmental changes such as aridification and eustatic changes. Mainland Australia has experienced significant environmental changes since the late Miocene, which heralded the onset of major aridification [24]. Other than a brief humid period during the Pliocene [25], this aridification intensified into the Pleistocene. While glacial periods in this region were not directly associated with the formation of glaciers, major changes in precipitation and temperature shifted ecosystems towards more arid conditions [26]. Concordantly, glacial maxima also drove eustatic changes, expanding much of the continental shelf as sea levels dropped [27]. The complex environmental history in southeast Australia, and its role on the evolution of temperate species, has been demonstrated by a number of phylogeographic studies (e.g. [28, 29]). For example, intense inland aridification during the Miocene and Pliocene has been associated with major coastward contractions in mesic species [30, 31], and rising post-glacial sea levels have driven the isolation of coastal and island populations of several terrestrial species (e.g., [27, 32, 33]).

Freshwater-dependent species are important indicators of historical environmental changes given their reliance on suitable habitat and often limited capacity for dispersal [34]. Within temperate southeast Australia, the often co-distributed southern (*Nannoperca australis*) and Yarra (*N. obscura*) pygmy perches provide an ideal comparative study system. Both species possess highly similar morphology, reproductive biology, salinity tolerance and habitat preferences, and also display similar patterns of metapopulation structure [35–39]. Both species have low dispersal capacity with little to nil contemporary connectivity among catchments [37, 39]. Both species are relatively old (e.g., their lineages diverged around 13 million years ago [40]) and show strong population structure, with two evolutionarily significant units (ESUs) separating coastal and inland (Murray-Darling Basin) populations in *N. australis* [28], and two clades each containing two ESUs in *N. obscura* [41]. Given their isolated populations, it is expected that their long-term persistence along landscapes depends on spatial variation of locally adaptive traits. This hypothesis is consistent with studies of *N. australis* that show that patterns of adaptation in traits related to reproductive fitness [42, 43], in levels of adaptive genetic diversity [38] and in variance of gene expression [44] are strongly associated with hydroclimatic gradients.

Despite their ecological similarities, the two species demonstrate marked differences in conservation status, genetic diversity and total distribution range. While both species are of conservation concern (*N. australis* as Near Threatened and *N. obscura* as Endangered) within the IUCN Red List [45] and in state conservation legislation, *N. obscura* is considered at higher risk due to their narrow range and extremely low genetic diversity [39, 46]. Substantial anthropogenic modification of riverine habitats throughout the Murray-Darling Basin since European colonisation ~ 200 years ago has likely exacerbated threats to the survival of both species [45, 47]. These factors are implicated in the local extirpation of *N. obscura* within the Murray-Darling Basin in the last 5 years [48], following failed reintroductions after a large-scale drought impacted the region [45]. The relatively low genetic diversity of *N. obscura* is not thought to be due to any particularly severe past bottleneck [46], complicating determining factors underlying this disparity. Additionally, it remains unclear whether the historical absence of *N. obscura* in some regions where *N. australis* is found is the result of historical local extinctions or a failure to initially colonise the habitat.

Here, we applied a comparative phylogeographic framework to explore the relative roles of ecological and demographic traits on evolutionary history. We used genomic datasets to estimate genetic diversity, phylogenetic relationships and demographic history of these two freshwater fishes, in conjunction with species distribution modelling. Then, we statistically evaluated regional concordance across co-occurring populations to assess whether the species shared demographic responses to Pleistocene glacial cycles. We predicted that evolutionary patterns, demographic histories and distribution changes would be concordant across the two species if ecological factors played a relatively strong role in determining species responses to past climatic changes, with current differences owing to different species responses to recent environmental changes. Contrastingly, discordant histories would indicate that genetic diversity and demography played a relatively larger role and underpinned their contemporary differences in conservation status. Our framework also includes differentiation of local-scale (population-level) and broad-scale (species-level) responses to assess the role of intraspecific patterns in driving lineage responses.

## Methods

### Sampling, genomic library preparation and ddRAD filtering

**Fig. 1:**
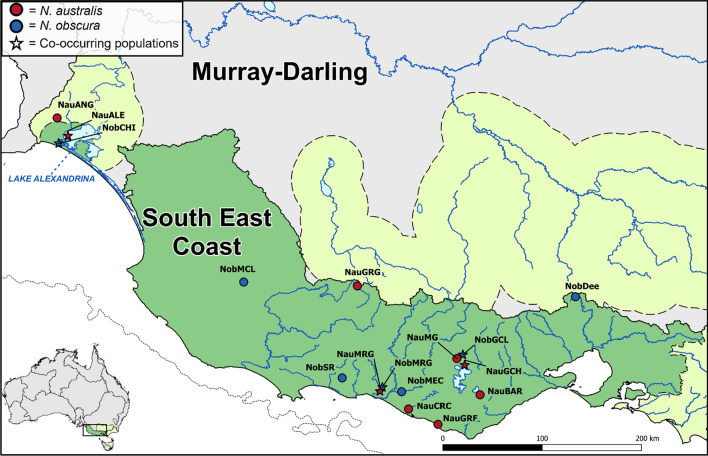
Contemporary distribution and sampling map for *N. australis* and *N. obscura*. *Nannoperca australis* sampling sites are indicated in red, and *N. obscura* sites in blue. The distribution of *N. australis* is indicated with light green shading and dashed borders, with the distribution of *N. obscura* (also the region of co-occurrence) in darker green. The solid black line indicates the boundary of major drainage basins, and the dotted line demonstrates the approximate shoreline during glacial maxima. Bottom left inset depicts study region and major drainage basins in Australia. Top right inset depicts the full extent of species distributions

While both species are distributed across southwest Victoria and the lower Murray-Darling Basin [35], *N. australis* is also found across eastern Victorian drainages, northern Tasmania and the highlands of the southern Murray-Darling Basin [49]. The final sample contains all known genetically distinct populations (including recently extirpated populations) across the species’ co-distributed range (Additional file 1: Table S1). This equals to seven populations of *N. obscura* and nine populations of *N. australis* occurring across all major drainages of the region (Fig. 1). An additional 10 and 15 N*. obscura* and *N. australis* (respectively) from Lake Alexandrina were also included for demographic analyses. For phylogenetic analyses, five samples of a sister species (*Nannoperca vittata*) were included as outgroup [40]. Specimens were collected using electrofishing, dip-, fyke- or seine-netting. Either the caudal fin or the entire specimen was stored at − 80 °C at the South Australian Museum, or in 99% ethanol at Flinders University.

DNA was extracted from muscle tissue or fin clips using a modified salting-out method [50] or a Qiagen DNeasy kit (Qiagen Inc., Valencia, CA, USA). Genomic DNA quality was checked using a spectrophotometer (NanoDrop, Thermo Scientific), 2% agarose gels, and a fluorometer (Qubit, Life Technologies). The ddRAD (double digest restriction-site associated DNA) genomic libraries were prepared in-house at the Molecular Ecology Lab of Flinders University following [38, 51], using the restriction enzymes *Sbf1* and *Mse1* and selecting fragments 300–800 base pairs in length. Our samples contain a combination of previously obtained sequences, from prior population genomic work on *N. australis* [38], phylogenomic work on all pygmy perches [40], and previously sequenced but unpublished samples. We supplemented these samples with additional sequences as part of a broader phylogeographic project (e.g., [49]; Additional file 1: Table S2). A total of 54 samples across seven *N. australis* (*n* = 33 samples) and four *N. obscura* (*n* = 21 samples) populations were previously paired-end sequenced on an Illumina HiSeq 2000 at Genome Quebec (Montreal, Canada). The remaining 44 samples were single-end sequenced on an Illumina HiSeq 2500 at the South Australia Health and Medical Research Institute (SAHMRI).

Sequences were demultiplexed using the ‘process_radtags’ module of Stacks 1.29 [52]. For paired-end sequences, only the forward read was retained, with all trimmed reads filtered and aligned using PyRAD 3.0.6 [53]. We used two separate alignment approaches: a species-wide alignment per species, with loci aligned across all populations per species (used for phylogenetic and historical migration analyses), as well as separate alignments for each population (used for demographic analyses). For species-wide alignments, only five representative samples per Lake Alexandrina population (NauALE and NobCHI) were used to prevent biasing loci towards these over-represented populations. Loci were retained if they occurred in at least ~ 80% of ingroup samples (22 in *N. obscura*; 31 in *N. australis;* Additional file 1: Methods). For individual population alignments, loci were re-aligned and SNPs called separately for each population (excluding those with *n* < 3) using PyRAD (Additional file 1: Methods). Only loci present in all individuals were kept to prevent missing data from biasing the site-frequency spectrum (SFS) in these individual population alignments.

### Contemporary genetic diversity

Population-level genetic diversity summaries (allelic richness and gene diversity) were estimated using the R package *hierfstat* [54]. Given uneven sample sizes, rarefaction was used (*n* = 4) to estimate mean values per locus per population. Due to the larger sample sizes available for Lake Alexandrina populations, genetic diversity parameters were also calculated using *n* = 15 rarefaction and loci aligned separately within each population. For these populations, we also calculated effective population size (*Ne*) using NeEstimator [55] and a minor allele frequency threshold of 0.02. Additionally, nucleotide diversity (π) within each population was estimated using dnaSP 6.1 [56]. Differences in population means of genetic diversity between the species were statistically evaluated using t-tests (two-tailed t-test or Wilcox test).

### Phylogenetic and historical migration analyses

Maximum likelihood (ML) phylogenies of each species were estimated using RAxML 8.2.11 [57] with the concatenated ddRAD alignments to estimate evolutionary relationships. Phylogenies were estimated under the GTR-GAMMA model of evolution and 1,000 RELL bootstraps for each species. Additionally, we estimated gene trees for each RAD locus using IQ-TREE2 [58] to account for genome-wide heterogeneity. Gene site concordance factors were estimated by comparing individual gene trees to the concatenated tree, and site concordance factors were calculated using 100 random quartets and the full species-wide concatenated alignments [59].

To better account for incomplete lineage sorting, we also estimated phylogenetic trees using the SNP-based multispecies coalescent approach SVDQuartets [60]. All unlinked SNPs from the species-wide alignments were used, with the most suitable substitution model first estimated using the automodel function of PAUP* 4 [61]. Trees were evaluated based on all possible quartets and 100 bootstrap replicates.

As historical migration may impact the topology of a phylogenetic tree, we also used TreeMix [62] to infer historical population connectivity. We iteratively increased the number of migrations from 0 to *n* for each species (nine in *N. australis;* seven in *N. obscura*) and evaluated the fit of each tree based on the standard error of the covariance matrix. We further assessed model fit by calculating the percentage of variance explained (https://github.com/wlz0726/Population_Genomics_Scripts/tree/master/03.treemix). The best supported number of migrations was determined by the asymptote of the likelihood, where additional migrations did not substantially increase model likelihood.

### Comparative demographic inference

Demographic histories for all populations were estimated using stairway plots and one-dimensional SFS calculated for each independent population alignment. Stairway plots were estimated assuming a mutation rate of 10^–8^ mutations per site per generation, and a generation time of one year for both species [46, 63]. Although both species reproduce annually, most individuals do not live beyond one to two years in the wild [63]. We then evaluated concordance of co-distributed populations under two coalescent frameworks; the populations of Gnarkeet Creek (NauGCH and NobGCL), Merri River (NauMRG and NobMRG) and Lake Alexandrina (NauALE and NobCHI) were selected based on their co-occurrence and to represent the geographic range of overlap in species distributions (Fig. 1).

We used FastSimCoal2 [64] to simulate model-based demographic histories over the last 30 Kyr. Simulations were conducted for each population under five different demographic scenarios (Additional file 1: Fig. S1). Parameters were estimated using 40 optimisation cycles with 500,000 simulations per scenario, with model fit estimated using Akaike Information Criterion and Akaike weights (Additional file 1: Methods and Table S3). Confidence intervals for the parameters of the best supported model per population were estimated by simulating 100 SFS and re-estimating parameters using 500,000 iterations per SFS.

Additionally, we ran co-demographic hierarchical approximate Bayesian computation simulations using the aggregate site frequency spectrum (aSFS) and Multi-DICE [65] to assess congruence. Since the aSFS requires equal sample sizes across the combined taxa, individual SFS for all populations were down-projected to the smallest population sample size (*n* = 8 haploid samples) using easySFS (github: isaacovercast/easySFS.git). A model of exponential growth followed by exponential decline was applied to all populations using broad uniform priors (Additional file 1: Methods and Fig. S2), based on results from FastSimCoal2. We first tested the proportion of co-contracting taxa (ξ), and then fixed this hyperparameter to better explore the remaining parameters. A “leave-one-out” approach using 50 pseudo-observed datasets was used to generate a confusion matrix, with the most likely ξ determined using the top 1,500 simulations and Bayes Factors. Parameters were estimated using 1.5 million simulations with posterior distributions estimated using the top 100 simulations and the *abc* R package [66]. We further tested for concordance across populations by comparing the posterior distributions for *Ne* and bottleneck strength (ε).

### Contemporary and paleoclimatic environmental modelling

Species distribution models (SDMs) were estimated using an ensemble modelling approach within biomod2 [67]. We estimated SDMs for both species across eleven time slices ranging from contemporary conditions to the Pliocene using the PaleoClim database [68]. Occurrence records for both species were obtained from a combination of sampled sites within this and past studies [28, 39, 40], and from the Atlas of Living Australia (http://www.ala.org.au/). We filtered the occurrence data to reduce the impact of spatial autocorrelation, resulting in final datasets of 1,021 and 163 observations for *N. australis* and *N. obscura,* respectively (Additional file 1: Methods and Fig. S3).

We selected eight non-correlated environmental variables for estimating species distributions (Additional file 1: Table S4). These were annual mean temperature (Bio1), mean diurnal range (Bio2), isothermality (Bio3), temperature seasonality (Bio6), mean temperature of the wettest quarter (Bio8), mean temperature of the driest quarter (Bio9), annual precipitation (Bio12) and precipitation seasonality (Bio15). For the three oldest time periods, Bio2, Bio3 and Bio6 were unavailable and thus not included within the projections. We generated three separate sets of pseudoabsences (*n* = 500) per species randomly from background cells > 50 km away from occurrences to reduce the likelihood of generating false absences within habitable areas [69]. Each dataset was replicated three times, with 80% of sites independently and randomly subset to train the model with presences and psuedoabsences equally weighted (prevalence = 0.5). SDMs were estimated for each dataset using the MaxEnt model, Random Forest (RF) and a generalised linear model (GLM; *n* = 27 models total), and an ensemble model generated per time period using the weighted mean of all models. These algorithms are among the most widely used for presence-only SDM analyses and encapsulate diverse statistical approaches, with the weighted mean the most commonly used ensemble generation approach [70]. All models were evaluated using both the relative operating characteristic and the true skill statistic. We quantitatively assessed the relative stability of species distributions over time by estimating the mean and standard deviation of suitability over time for each species. Differences in distributional ranges between species across time were estimated by converting SDMs to binary presence-absence maps (Additional file 1: Methods).

## Results

### Bioinformatics

We obtained 21,051 ddRAD loci containing 53,334 filtered SNPs for *N. obscura* and 19,428 ddRAD loci containing 69,264 filtered SNPs for *N. australis*, with low missing data in both alignments (Additional file 1: Fig. S4)***.*** Genetic diversity differed remarkably between the two species, with allelic richness, gene diversity, nucleotide diversity and number of SNPs per population alignment being significantly higher (p ≤ 0.01) in *N. australis* (Table 1).

**Table 1:** Population genetic summaries of *N. australis* and *N. obscura*

Alignment			Species-wide		Individual populations		
Species	Pop	N	AR	Hs	Ne	Π	SNPs
*N. australis*	NauANG	5	1.138 (± 0.339)	0.029 (± 0.106)		1.59 × 10^–4^	2,446
	NauALE	5 20	1.354 (± 0.480)	0.062 (± 0.165) 0.179 (± 0.142)	71.3 [35.9–520.9]	1.23 × 10^–3^	1198
	NauGRG	5	1.123 (± 0.313)	0.035 (± 0.108)		8.84 × 10^–5^	5282
	NauMRG	5	1.116 (± 0.312)	0.037 (± 0.119)		1.62 × 10^–4^	3835
	NauGRF	5	1.092 (± 0.285)	0.027 (± 0.106)		7.84 × 10^–5^	2969
	NauBAR	4	1.240 (± 0.428)	0.061 (± 0.145)		2.02 × 10^–4^	5084
	NauMG	4	1.216 (± 0.413)	0.056 (± 0.138)		1.57 × 10^–4^	5357
	NauGCH	4	1.181 (± 0.387)	0.047 (± 0.136)		1.88 × 10^–4^	4272
	Total	56	1.241	0.0439		2.83 × 10^–4^	17,389
*N. obscura*	NobCHI	5 15	1.151 (± 0.347)	0.038 (± 0.117) 0.136 (± 0.108)	14.9 [4.6–652.2]	1.69 × 10^–4^	721
	NobMEC	5	1.030 (± 0.166	0.011 (± 0.069)		2.11 × 10^–5^	1002
	NobMCL	4	1.100 (± 0.290)	0.026 (± 0.094)		4.18 × 10^–5^	1633
	NobMRG	4	1.058 (± 0.234)	0.020 (± 0.093)		3.23 × 10^–5^	1454
	NobGCL	5	1.070 (± 0.250)	0.019 (± 0.088)		2.49 × 10^–5^	1350
	Total	33	1.168	0.0237		5.78 × 10^–5^	15,715
T-test			T = 2.38 (*p* < 0.04)	T = 2.93 (*p* = 0.01)		W = 56 (*p* = 0.01)	T = 4.63 (*p* < 0.01)

N = total number of samples per population. AR = rarefied allelic richness. Hs = rarefied gene diversity. *Ne* = effective population size, with 95% confidence intervals estimated by jack-knifing in square brackets. π = nucleotide diversity. Species-wide alignment values are reported as means of means across all loci ± standard deviation under rarefaction (*n* = 4). For Lake Alexandrina populations, gene diversities were also calculated for individual population alignments and rarefaction of *n* = 15 samples (reported second). Populations means across species were compared using either an unpaired two-samples t-test (T) or Wilcoxon rank test (W)

### Phylogenetic analysis

**Fig. 2:**
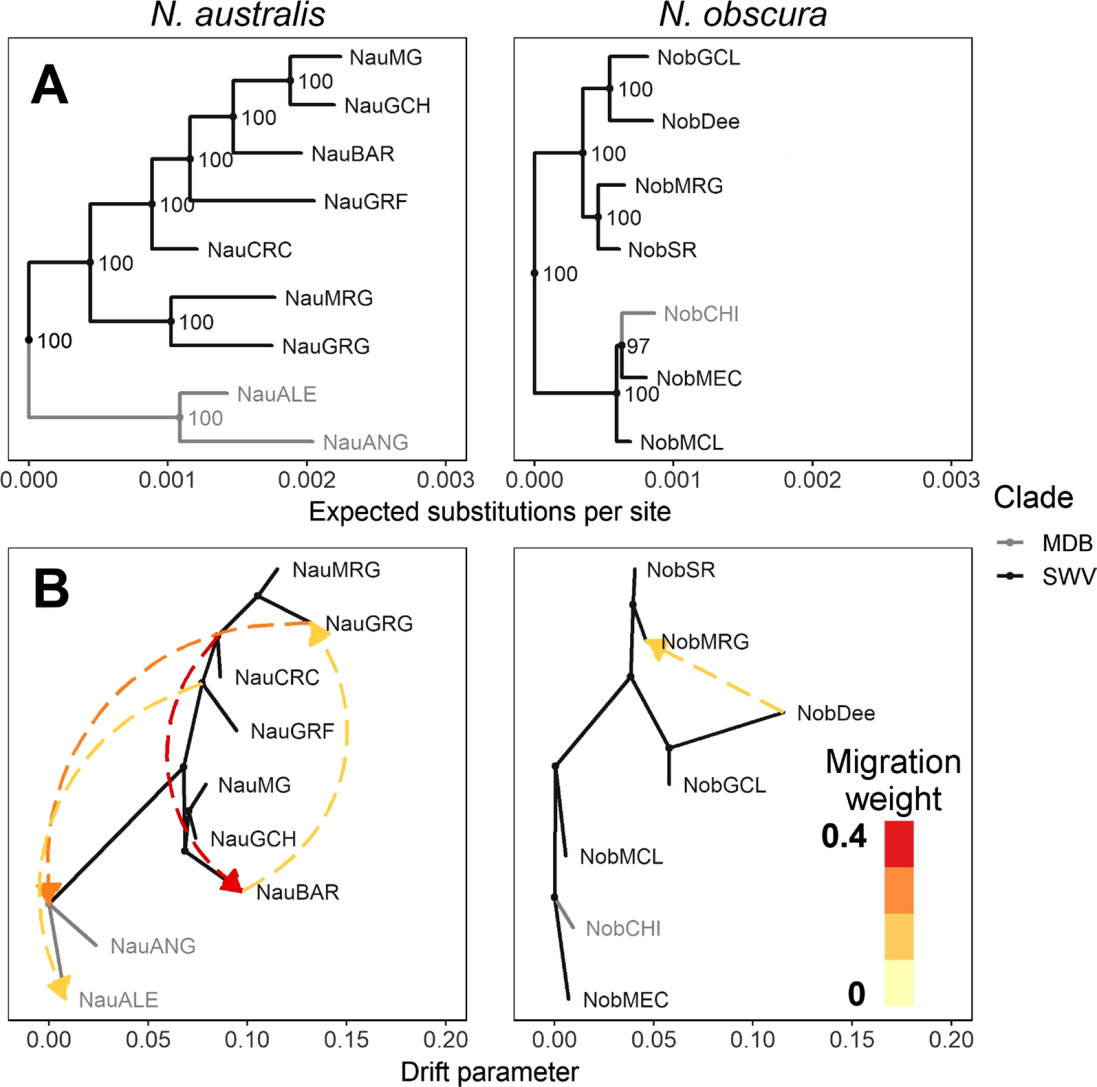
Phylogenetic histories and migration patterns in *N. australis* and *N. obscura.* **A** Maximum likelihood phylogenetic trees based on ddRAD loci. Populations were reciprocally monophyletic and so were collapsed to the population level for simplicity. Both trees were rooted using *N. vittata* as the outgroup, which was dropped for visualisation. Node values show bootstrap support. Branch colours indicate the drainage basin of origin for each population or clade. **B** Best supported ancestral migration patterns inferred using TreeMix based on SNP datasets. All displayed migrations were statistically significant (p < 0.05). Arrows denote the direction of inferred migrations, with the colour indicating their relative weights

Phylogenetic analysis of both datasets returned a highly supported phylogenetic tree for each species. Species trees estimated using SVDQuartets were concordant with the maximum likelihood trees, with strong support all for major nodes (> 70%; Additional file 1: Fig. S5). Site concordance factors broadly supported these patterns, although gene concordance factors were low across both trees (Additional file 1: Figs. S6–S8)—this is not unexpected when gene trees are estimated from short and relatively uninformative individual loci [59]. For southern pygmy perch, the topology of this phylogenetic tree mirrored the geographic range of the samples, with a clear division between the Murray-Darling Basin ESU and the coastal ESU within the tree (Fig. 2A). Within the coastal clade, populations diverged in a longitudinal manner, with eastern populations as the most recently diverged. In contrast, the phylogenetic tree for *N. obscura* did not demonstrate the same precise patterns, with populations not diverging in an exactly longitudinal manner. However, this was driven by a single outlier population (NobMEC).

TreeMix inferred a greater number of migration events within *N. australis* (four) than *N. obscura* (one event) (Fig. 2B; Additional file 1: Fig. S9). Within *N. australis*, migrations were inferred both across populations of the coastal lineage as well as into the Murray-Darling Basin. The strongest migrations were between eastern coastal populations, and from the ancestor of the westernmost coastal population into the ancestor of the Murray-Darling Basin lineages. For *N. obscura*, the single migration inferred suggested historical gene flow from the easternmost population to a more central population. Trees and migration edges for both species were well supported by covariance matrices, with low pairwise residuals (Additional file 1: Fig. S10) and standard errors of < 1 for any given population for both species (Additional file 1: Fig. S11).

### Comparative demography

**Fig. 3:**
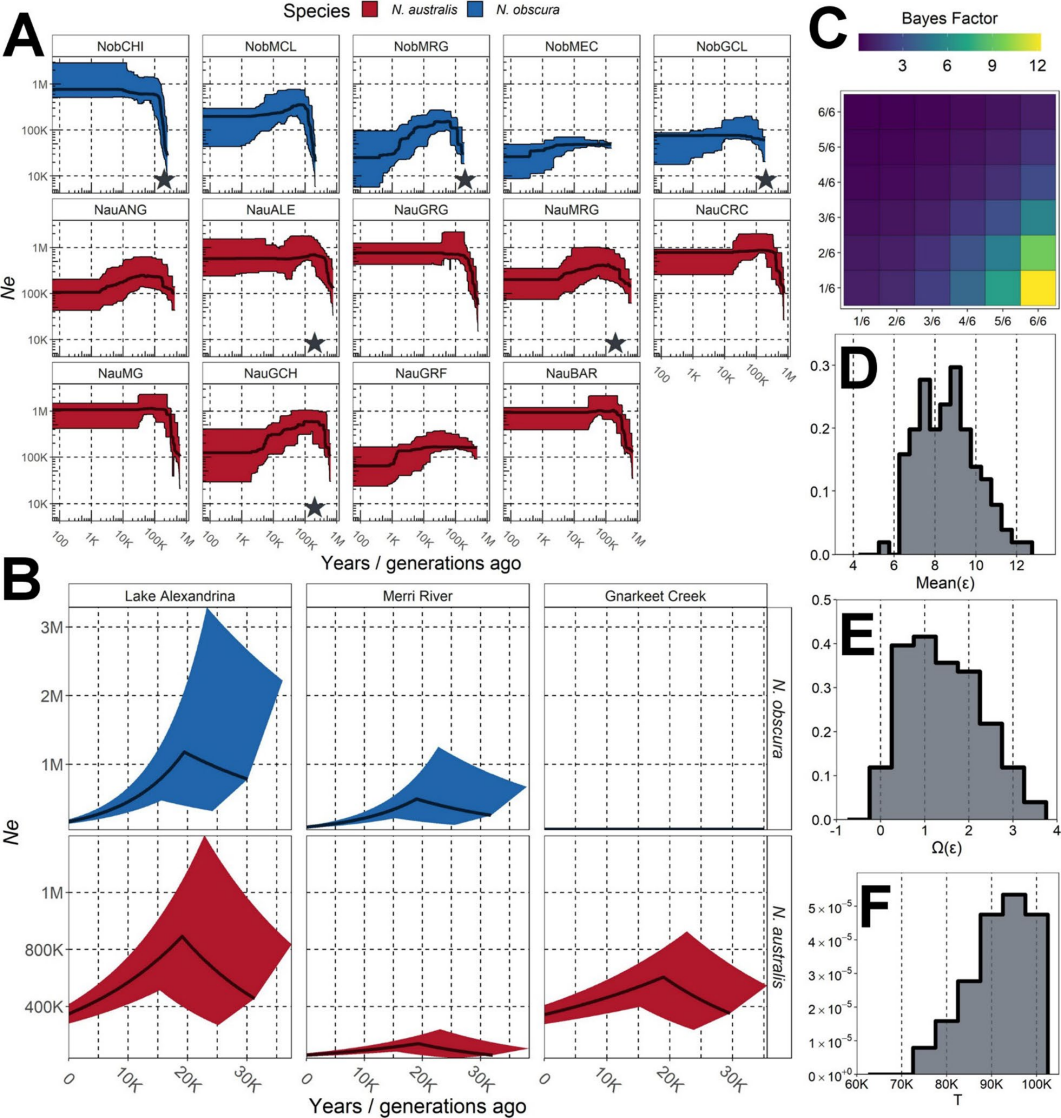
Demographic histories of *N. australis* and *N. obscura* populations. **A** Stairway plot reconstructions of demographic history. Inset stars indicate co-occurring populations which were further explored within a codemographic framework. Populations are arranged from westernmost to easternmost within each species. **B** Most likely individual demographic histories for co-occurring *N. australis* and *N. obscura* populations over the Pleistocene, simulated using FastSimCoal2. Thick dark lines indicate mean *Ne* over time, calculated based on the means of current *Ne*, rates of change and timing of switching rates (see Supplementary Material). Shaded areas indicate 95% confidence intervals based on the 97.5% and 2.5% probability estimates for the same parameters. **C** Bayes Factor matrix of the proportion of populations showing synchronised bottlenecks (ξ) within a co-demographic model using Multi-DICE. Each cell compares the model in the column with the model in the row, with brighter colours indicating greater support for the column. **D** Posterior distribution of mean bottleneck strength (ε) across all six populations. **E** Posterior distribution of dispersion index of bottleneck strength [Var(ε)/Mean(ε)] across all six populations. **F** Posterior distribution of the timing of the bottleneck event, in generations/years

Stairway plots demonstrated broadly similar demographic histories across the two species, with most populations relatively stable or declining slightly over the last 1 Mya (Fig. 3A). Populations within both species demonstrated variable demographic histories, although populations of *N. australis* appeared generally more stable over time. Both Lake Alexandrina populations (NobCHI and NauALE) showed significant historical increases in *Ne* > 200 Kya, and long-term stable population sizes following this expansion.

Most populations chosen for comparative analysis demonstrated fluctuating demographic histories (Fig. 3B), with a period of pre-LGM (Last Glacial Maximum) growth followed by a post-LGM decline (Additional file 1: Table S5). Only the eastern *N. obscura* population (NobGCL) contrasted this pattern, with a model of low but constant population size more supported than other demographic histories (Model 3). Strong post-glacial declines were present in Lake Alexandrina populations of both species, with weaker declines in the more eastern population pairs.

A confusion matrix suggested that the co-demographic model was more likely to infer fully synchronous (ξ = 1) or fully asynchronous (ξ = 0.167) co-contractions over intermediate proportions of taxa (Additional file 1: Fig. S12). Despite this, Bayes Factors supported a fully synchronous model over more asynchronous models, and so ξ was fixed to 1 to better explore other parameters (Fig. 3C). Contemporary population sizes were inferred to be relatively small across all populations with relatively weak post-glacial bottleneck strength (Fig. 3D; Additional file 1: Table S6). These bottlenecks were similar in magnitude across populations, as indicated by low values of the dispersion index (Fig. 3E). However, Multi-DICE did not recover the same timing of the bottleneck, possibly due to relatively low resolution within the aSFS (Fig. 3F). Overall, these results support a widespread and concordant bottleneck across the six co-distributed populations.

### Species distribution modelling

**Fig. 4:**
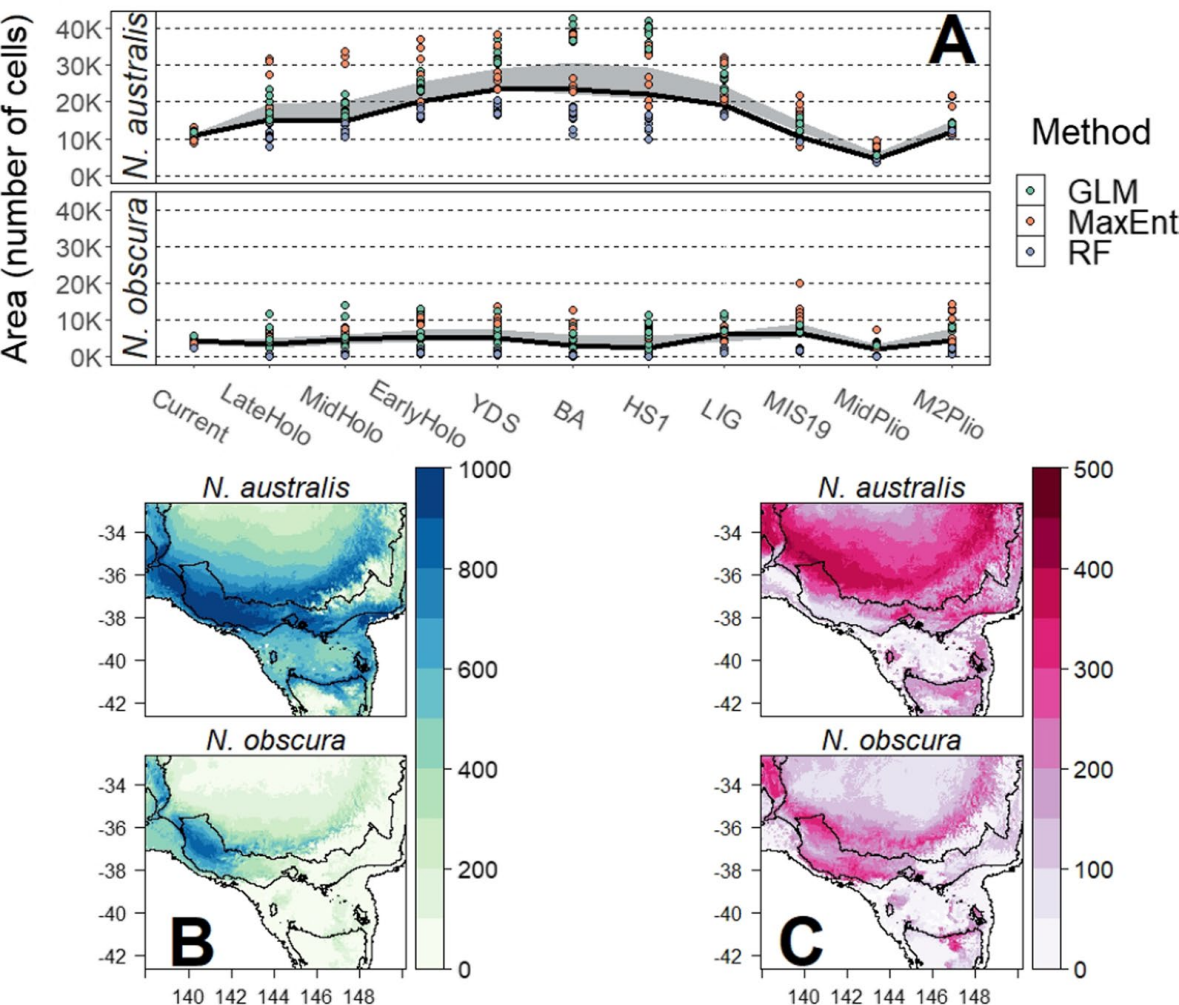
Comparisons of summaries of distributional changes over eleven time periods spanning the Plio-Pleistocene. **A** Distribution extent per species. Individual models are indicated by points, with SDM method indicated by colour. The 95% confidence interval across all individual models is shown by the pale blue ribbon. The ensemble model is represented by a solid black line. **B** Mean cell suitability across all time periods. **C** Variation (standard deviation) in cell suitability across all time periods

Comparing the SDMs of the two species indicated much greater maximum distribution and variation in distributional range in *N. australis* than in *N. obscura*. *Nannoperca obscura* demonstrated likely long-term isolation to a relatively small region of southwest Victoria, whilst *N. australis* demonstrated a potentially significant range expansion event throughout the early Pleistocene with a more recent contraction in the Holocene (Additional file 1: Fig. S13). Despite these differences, both species were suggested to have maintained a putative shared climatic refugium in southwest Victoria, highlighted by a region of high mean suitability in both species (Fig. 4B).

Comparisons across the different methods indicated that RandomForest was more conservative in estimating area (Fig. 4A). While there was significant variation in estimated area across the different methods, ensemble models approximately captured the mean of all models. Historical projections suggested that *N. australis* had a significantly larger distribution throughout the Pleistocene compared to the relatively stable range of *N. obscura*, with the former projected to have spanned a range approximately twice as large as the latter during the mid-Pleistocene (Fig. 4A). These patterns were similarly reflected within the standard deviations across timeslices per species, with *N. australis* showing much higher variation over a larger area (Fig. 4C).

## Discussion

Our results demonstrate how spatial variation in demographic history between species may drive discordant responses to past climatic changes in parts of their range, even when local-scale impacts are concordant and species’ ecological traits are similar. Specifically, we show that within a projected shared climatic refugium for two co-distributed and ecologically similar freshwater fishes, demographic histories were largely concordant. However, towards the edges of this refugium demographic histories decreased in concordance, suggesting that range edge populations of *N. obscura* were more limited than *N. australis* in their capacity for expansion during more favourable climatic conditions. Together, our findings determine the importance of intraspecific, population-level dynamics in driving species-wide adaptation and resilience to climate change.

The temperate zone of southeast Australia has undergone significant environmental change since the Pliocene, owing to a combination of continent-wide aridification [24], eustatic changes [28] and major hydrological rearrangements [71]. These various aspects likely had significant impacts on the persistence and connectivity of freshwater lineages across the region [49]. This was supported by the high level of phylogenetic structure within *N. australis,* and the inferred migration pathways that correspond well to those previously suggested through ancient hydrological conduits [71]. Although phylogenetic patterns in *N. obscura* did not directly match the longitudinal gradient of populations, earlier phylogenetic analyses using allozymes and mitochondrial DNA showed a similar pattern [41]. This disjunction was attributed to potential historical connections from Mount Emu Creek into more western populations [39], although short branch lengths and low genetic diversity may also indicate incomplete lineage sorting as a factor [72]. For both species, we denote two major clades: one of Murray-Darling Basin populations and another of coastal populations in *N. australis*, as suggested elsewhere [49], and two clades each containing two previously identified ESUs in *N. obscura* [41].

Within the species distribution models, a region of southwest Victoria was suggested as a potential climatic refugium for both species throughout the Plio-Pleistocene. This region was consistently suggested as suitable habitat for both species across all time slices. Although glacial maxima were associated with cold and arid conditions across Australia, coastal woodland habitats were likely buffered against intense aridification by oceanic circulation and relatively higher humidity and rainfall [73]. Other phylogeographical studies demonstrating limited impact of glacial maxima on connectivity supports the identity of this climatic refugium [27, 73]. Co-occurring populations within this shared refugium demonstrated highly congruent demographic histories at both more ancient (> 1 Myr) and more recent (since the LGM) temporal scales. This concordance is expected when ecological traits, habitat preferences and environmental stability are shared across the species in question [21]. Although individual populations within each species demonstrated spatially variable demographic histories, comparisons across the two species showed similar patterns of *Ne* over time for most directly co-occurring populations.

Both pygmy perch species demonstrated synchronous expansions during the LGM with post-glacial contractions across central populations. Despite intense inland aridification during glacial maxima, run-off in many southeast Australian rivers were likely much greater during the LGM [74]. These increased river flows have been attributed to seasonal snow melt of periglacial regions in the highlands and reduced vegetation cover, creating large rivers with enhanced run-off [31, 74]. Colder conditions and strong flows may have facilitated the observed concordant expansion in populations at this time, with the steep decline in flows during the early Holocene (14–7 Kya) potentially contributing to their more recent contraction [75]. However, concordance was reduced for pairwise populations that occurred closer to the edge of this shared refugium, suggesting the species had discordant responses at the fringe of the range. Similarly, phylogenetic patterns at the species-wide level varied between the two species, with clearer geographic sorting and historical migration across *N. australis* lineages compared to *N. obscura*.

Spatial variation in demographic history, and by extension concordance across taxa, may result from several different mechanisms [22, 76]. Particularly for narrowly distributed species, edge-of-range effects on populations close to the ecological tolerance threshold of the species may result in highly divergent patterns of demographic history and genetic diversity compared to more central populations [12, 76]. By extension, the ecological range of species may be a strong factor driving discordance when particular locations are at the periphery of the distribution of one species, but not another. Given the broad similarity in ecological traits between the two species and their co-occurring nature [77], it is unlikely that this discordance in species-wide responses to past climatic changes is a result of different ecologies. However, some variation in microhabitat preference seems to exist between species, with *N. obscura* limited to larger, lowland channels and floodplains whereas *N. australis* is also found in streams and dense swamps [77]. This suggests greater habitat specialisation in *N. obscura*, which might drive lower SGV (or result from it) and impede range expansions. Thus, we cannot completely rule out some role of ecology and its interactions with genetic diversity in driving discordant responses. The lower genetic diversity in *N. obscura* could not be directly attributed to notable and widespread genetic bottlenecks, suggesting instead that the species suffered from a consistent pattern of being genetically depauperate. Combined, these factors suggest that long-term SGV may be a key factor driving the temporally and spatially widespread discordance in response to Pleistocene climate changes.

Adaptive responses, particularly in scenarios of range expansion, are often driven by soft sweeps of SGV [8]. While many studies focus on rapid adaptation from SGV in terms of invasive species colonising new habitats [78], similar dynamics can be expected to play a role in range expansions of native taxa [79, 80]. In regard to range shifts across the Pleistocene, higher SGV may have predisposed *N. australis* to capitalise on the colder temperatures and stronger rivers of glacial periods and subsequently expand. Similarly, historical connectivity across now-isolated river drainages [28] likely facilitated interpopulation gene flow, which may have further bolstered SGV and adaptive potential [76, 80]. This gene flow in *N. australis* may have also facilitated range expansion if locally adaptive alleles were transferred into edge populations [78]. Contrastingly, a lack of long-term SGV within *N. obscura* may have prevented them from expanding under these conditions, leading to the species-wide discordance. The spatial variation in the degree of concordance, with discordance occurring at the edge of the *N. obscura* pre-glacial refugium, supports this conclusion.

Discordant species-wide responses to past climatic change may play an important role in contemporary genetic diversity and, by extension, current conservation efforts. For example, low genetic diversity resulting from historical bottlenecks can drive contemporary inbreeding depression [81]. Additionally, the parallels between historical range expansion scenarios and current reintroductions to conserve species demonstrates how historical processes may inform current practices [82]. For example, reduced adaptive capacity in *N. obscura* may have contributed to their local extirpation and to the failure of reintroductions of captive-born offspring at range margins, as documented for the lower Murray-Darling Basin [45]. This contrasts to the successful reintroduction of *N. australis* that simultaneously took place in that site using the same captive-breeding design [45, 46].

Understanding how, and which, species may be able to adapt under contemporary climate change remains a critical aspect of evolutionary biology [2]. Typically, this framework has focused on understanding how ecological traits may underpin individual species responses to climatic change [6]. However, demographic parameters are also critical components for species susceptibility to contemporary climate change [5]. Here, we demonstrate that intraspecific SGV may also be a critical component of species responses to climatic changes, particularly in range-edge populations. This corroborates studies indicating that adaptive potential is largely driven by SGV prior to the origination of major selective pressure [9] and suggests that considering broad ecology alone may not be enough to predict species’ ability to respond. Thus, understanding how the demographic history of individual populations may predispose, or hinder, species adaptive potential is an important component of conservation management of threatened species. For species with low SGV, proactive measures such as assisted gene flow and maintenance of effective population size may assist in their long-term conservation [83].

## Conclusion

Differences in long-term standing genetic variation drove discordance in the response of closely related and ecologically similar freshwater fishes to historical climate change, by potentially facilitating range expansion of one species but not the other. However, in the centre of a putatively shared habitat refugium, demographic histories were concordant, suggesting that spatial variation in the degree of concordance is linked to the interaction of standing genetic variation and distribution edge effects. Together, this demonstrates the importance of the maintenance of standing genetic variation for adaptive potential in response to climatic changes and the role of non-ecological traits in driving patterns of concordance or discordance.

## Supplementary Information

**Additional file 1: Methods.** Additional details of sequencing, demographic modelling and species distribution modelling methods. **Table S1.** Locality data for samples used in this study. **Table S2**. Breakdown of sequencing approach for all samples. **Table S3**. Prior ranges for individual population demographic models estimated within FastSimCoal2. **Table S4**. Pearson’s pairwise correlation for all 19 bioclimatic variables. **Table S5.** Model likelihoods for demographic syndrome models estimated using FastSimCoal2. **Table S6**. Posterior distributions of parameters from co-demographic models in Multi-DICE. **Figure S1**. Diagrammatic representations of demographic syndromes tested per population selected for codemographic analysis, using FastSimCoal2. **Figure S2**. Diagrammatic representations of codemographic models within Multi-DICE. **Figure S3.** Maps of occurrence data used for species distribution modelling. **Figure S4**. Histogram of missing data per sample in the species-wide alignments. **Figure S5.** Unrooted species trees inferred using SVDQuartets. **Figure S6**. Full maximum likelihood phylogenetic tree for *N. australis*. **Figure S7**. Full maximum likelihood phylogenetic tree for *N. obscura.* **Figure S8**. Gene and site concordance factors between gene trees estimated with individual RAD loci and the concatenated phylogenies. **Figure S9**. Likelihoods and percentage of variance explained in TreeMix models. **Figure S10**. Residual matrices under the best supported TreeMix models. **Figure S11**. Standard error of the covariance matrices under the best supported TreeMix models. **Figure S12**. Confusion matrix of ξ hyperparameter within the co-demographic Multi-DICE model. **Figure S13**. Ensemble species distribution models over time.

## Data Availability

The sequence alignment and SNP datasets generated and analysed during this study, as well as the occurrence data used to estimate species distribution models, are available in Figshare (https://doi.org/10.25451/flinders.18318602).

## References

[1] Waldvogel AM, Feldmeyer B, Rolshausen G, Exposito-Alonso M, Rellstab C, Kofler R, Mock T, Schmid K, Schmitt I, Bataillon T (2020). Evolutionary genomics can improve prediction of species’ responses to climate change. Evol Lett.

[2] Healy TM, Brennan RS, Whitehead A, Schulte PM (2018). Tolerance traits related to climate change resilience are independent and polygenic. Global Change Biol.

[3] Somero GN (2010). The physiology of climate change: how potentials for acclimatization and genetic adaptation will determine ‘winners’ and ‘losers’. J Exp Biol.

[4] Travis JMJ, Delgado M, Bocedi G, Baguette M, Bartoń K, Bonte D, Boulangeat I, Hodgson JA, Kubisch A, Penteriani V (2013). Dispersal and species’ responses to climate change. Oikos.

[5] Pearson RG, Stanton JC, Shoemaker KT, Aiello-Lammens ME, Ersts PJ, Horning N, Fordham DA, Raxworthy CJ, Ryu HY, McNees J (2014). Life history and spatial traits predict extinction risk due to climate change. Nat Clim Chang.

[6] Williams SE, Shoo LP, Isaac JL, Hoffmann AA, Langham G (2008). Towards an integrated framework for assessing the vulnerability of species to climate change. PLoS Biol.

[7] Hoffmann AA, Sgro CM (2011). Climate change and evolutionary adaptation. Nature.

[8] Stange M, Barrett RDH, Hendry AP (2021). The importance of genomic variation for biodiversity, ecosystems and people. Nat Rev Genet.

[9] Lai Y-T, Yeung CKL, Omland KE, Pang E-L, Hao Y, Liao B-Y, Cao H-F, Zhang B-W, Yeh C-F, Hung C-M (2019). Standing genetic variation as the predominant source for adaptation of a songbird. PNAS.

[10] Morris MRJ, Bowles E, Allen BE, Jamniczky HA, Rogers SM (2018). Contemporary ancestor? Adaptive divergence from standing genetic variation in Pacific marine threespine stickleback. BMC Evol Biol.

[11] DeWoody JA, Harder AM, Mathur S, Willoughby JR (2021). The long-standing significance of genetic diversity in conservation. Mol Ecol.

[12] Eckert CG, Samis KE, Lougheed SC (2008). Genetic variation across species’ geographical ranges: the central-marginal hypothesis and beyond. Mol Ecol.

[13] Smith S, Brauer CJ, Sasaki M, Unmack PJ, Guillot G, Laporte M, Bernatchez L, Beheregaray LB (2020). Latitudinal variation in climate-associated genes imperils range edge populations. Mol Ecol.

[14] Polechova J, Barton NH (2015). Limits to adaptation along environmental gradients. PNAS.

[15] Bridle JR, Polechova J, Kawata M, Butlin RK (2010). Why is adaptation prevented at ecological margins? New insights from individual-based simulations. Ecol Lett.

[16] Angert AL, Bradshaw HD, Schemske DW (2008). Using experimental evolution to investigate geographic range limits in monkeyflowers. Evolution.

[17] Fordham DA, Brook BW, Moritz C, Nogues-Bravo D (2014). Better forecasts of range dynamics using genetic data. Trends Ecol Evol.

[18] Potter S, Xue AT, Bragg JG, Rosauer DF, Roycroft EJ, Moritz C (2018). Pleistocene climatic changes drive diversification across a tropical savanna. Mol Ecol.

[19] Paz A, Ibanez R, Lips KR, Crawford AJ (2015). Testing the role of ecology and life history in structuring genetic variation across a landscape: a trait-based phylogeographic approach. Mol Ecol.

[20] Avise JC, Bowen BW, Ayala FJ (2016). In the light of evolution X: comparative phylogeography. PNAS.

[21] Zamudio KR, Bell RC, Mason NA (2016). Phenotypes in phylogeography: species’ traits, environmental variation, and vertebrate diversification. PNAS.

[22] Papadopoulou A, Knowles LL (2016). Toward a paradigm shift in comparative phylogeography driven by trait-based hypotheses. PNAS.

[23] DeChaine EG, Martin AP (2005). Historical biogeography of two alpine butterflies in the Rocky Mountains: broad-scale concordance and local-scale discordance. J Biogeogr.

[24] Byrne M, Yeates DK, Joseph L, Kearney M, Bowler J, Williams MA, Cooper S, Donnellan SC, Keogh JS, Leys R (2008). Birth of a biome: insights into the assembly and maintenance of the Australian arid zone biota. Mol Ecol.

[25] McLaren S, Wallace MW (2010). Plio-Pleistocene climate change and the onset of aridity in southeastern Australia. Global Planet Change.

[26] Duckett PE, Stow AJ, Burridge C (2013). Higher genetic diversity is associated with stable water refugia for a gecko with a wide distribution in arid Australia. Divers Distrib.

[27] Schultz MB, Ierodiaconou DA, Smith SA, Horwitz P, Richardson AM, Crandall KA, Austin CM (2008). Sea-level changes and palaeo-ranges: reconstruction of ancient shorelines and river drainages and the phylogeography of the Australian land crayfish *Engaeus sericatus* Clark (Decapoda: Parastacidae). Mol Ecol.

[28] Unmack PJ, Hammer MP, Adams M, Johnson JB, Dowling TE (2013). The role of continental shelf width in determining freshwater phylogeographic patterns in south-eastern Australian pygmy perches (Teleostei: Percichthyidae). Mol Ecol.

[29] Neal WC, James EA, Bayly MJ (2019). Phylogeography, classification and conservation of pink zieria (*Zieria veronicea*; Rutaceae): influence of changes in climate, geology and sea level in south-eastern Australia. Plant Syst Evol.

[30] Byrne M, Steane DA, Joseph L, Yeates DK, Jordan GJ, Crayn D, Aplin K, Cantrill DJ, Cook LG, Crisp MD (2011). Decline of a biome: evolution, contraction, fragmentation, extinction and invasion of the Australian mesic zone biota. J Biogeogr.

[31] Pepper M, Keogh JS (2021). Life in the “dead heart” of Australia: The geohistory of the Australian deserts and its impact on genetic diversity of arid zone lizards. J Biogeogr.

[32] Norgate M, Chamings J, Pavlova A, Bull JK, Murray ND, Sunnucks P (2009). Mitochondrial DNA indicates late Pleistocene divergence of populations of *Heteronympha merope*, an emerging model in environmental change biology. PLoS ONE.

[33] Kreger KM, Shaban B, Wapstra E, Burridge CP (2020). Phylogeographic parallelism: concordant patterns in closely related species illuminate underlying mechanisms in the historically glaciated Tasmanian landscape. J Biogeogr.

[34] Davis CD, Epps CW, Flitcroft RL, Banks MA (2018). Refining and defining riverscape genetics: how rivers influence population genetic structure. Wiley Interdiscip Rev Water.

[35] Wedderburn SD, Hammer MP, Bice CM (2012). Shifts in small-bodied fish assemblages resulting from drought-induced water level recession in terminating lakes of the Murray-Darling Basin, Australia. Hydrobiologia.

[36] Hammer MP, Bice CM, Hall A, Frears A, Watt A, Whiterod NS, Beheregaray LB, Harris JO, Zampatti BP (2013). Freshwater fish conservation in the face of critical water shortages in the southern Murray-Darling Basin, Australia. Mar Freshw Res.

[37] Cole TL, Hammer MP, Unmack PJ, Teske PR, Brauer CJ, Adams M, Beheregaray LB (2016). Range-wide fragmentation in a threatened fish associated with post-European settlement modification in the Murray-Darling Basin. Australia Conserv Genet.

[38] Brauer CJ, Hammer MP, Beheregaray LB (2016). Riverscape genomics of a threatened fish across a hydroclimatically heterogeneous river basin. Mol Ecol.

[39] Brauer CJ, Unmack PJ, Hammer MP, Adams M, Beheregaray LB (2013). Catchment-scale conservation units identified for the threatened Yarra pygmy perch (*Nannoperca obscura*) in highly modified river systems. PLoS ONE.

[40] Buckley SJ, Domingos FMCB, Attard C, Brauer CJ, Sandoval-Castillo J, Lodge R, Unmack P, Beheregaray LB (2018). Phylogenomic history of enigmatic pygmy perches: implications for biogeography, taxonomy and conservation. Royal Soc Open Sci..

[41] Hammer MP, Unmack PJ, Adams M, Johnson JB, Walker KF (2010). Phylogeographic structure in the threatened Yarra pygmy perch *Nannoperca obscura* (Teleostei: Percichthyidae) has major implications for declining populations. Conserv Genet.

[42] Morrongiello JR, Bond NR, Crook DA, Wong BB (2010). Nuptial coloration varies with ambient light environment in a freshwater fish. J Evol Biol.

[43] Morrongiello JR, Bond NR, Crook DA, Wong BBM (2012). Spatial variation in egg size and egg number reflects trade-offs and bet-hedging in a freshwater fish. J Anim Ecol.

[44] Brauer CJ, Unmack PJ, Beheregaray LB (2017). Comparative ecological transcriptomics and the contribution of gene expression to the evolutionary potential of a threatened fish. Mol Ecol.

[45] Beheregaray LB, Attard CR, Brauer CJ, Whiterod NS, Wedderburn SD, Hammer MP. Conservation breeding and reintroduction of pygmy perches in the lower Murray-Darling Basin, Australia: two similar species, two contrasting outcomes. In: Soorae PS, editor. Global conservation translocation perspectives: 2021 Case studies from around the globe*.* Gland, Switzerland; IUCN SSC Conservation Translocation Specialist Group, Environment Agency, 2021.p. 26–31.

[46] Attard CR, Moller LM, Sasaki M, Hammer MP, Bice CM, Brauer CJ, Carvalho DC, Harris JO, Beheregaray LB (2016). A novel holistic framework for genetic-based captive-breeding and reintroduction programs. Conserv Biol.

[47] Brauer CJ, Beheregaray LB (2020). Recent and rapid anthropogenic habitat fragmentation increases extinction risk for freshwater biodiversity. Evol Appl.

[48] Wedderburn SD, Whiterod NS, Vilizzi L (2021). Occupancy modelling confirms the first extirpation of a freshwater fish from one of the world's largest river systems. Aquat Conserv Mar Freshwat Ecosyst.

[49] Buckley SJ, Brauer C, Unmack PJ, Hammer MP, Beheregaray LB (2021). The roles of aridification and sea level changes in the diversification and persistence of freshwater fish lineages. Mol Ecol.

[50] Sunnucks P, Hales DF (1996). Numerous transposed sequences of mitochondrial cytochrome oxidase I-II in aphids of the genus *Sitobion* (Hemiptera: Aphididae). Mol Biol Evol.

[51] Peterson BK, Weber JN, Kay EH, Fisher HS, Hoekstra HE (2012). Double digest RADseq: an inexpensive method for *de novo* SNP discovery and genotyping in model and non-model species. PLoS ONE.

[52] Catchen J, Hohenlohe PA, Bassham S, Amores A, Cresko WA (2013). Stacks: an analysis tool set for population genomics. Mol Ecol.

[53] Eaton DA (2014). PyRAD: assembly of de novo RADseq loci for phylogenetic analyses. Bioinformatics.

[54] Goudet J (2005). hierfstat, a package for r to compute and test hierarchical F-statistics. Mol Ecol Notes.

[55] Do C, Waples RS, Peel D, Macbeth GM, Tillett BJ, Ovenden JR (2014). NeEstimator v2: re-implementation of software for the estimation of contemporary effective population size (Ne ) from genetic data. Mol Ecol Resour.

[56] Rozas J, Ferrer-Mata A, Sanchez-DelBarrio JC, Guirao-Rico S, Librado P, Ramos-Onsins SE, Sanchez-Gracia A (2017). DnaSP 6: DNA sequence polymorphism analysis of large data sets. Mol Biol Evol.

[57] Stamatakis A (2014). RAxML version 8: a tool for phylogenetic analysis and post-analysis of large phylogenies. Bioinformatics.

[58] Minh BQ, Schmidt HA, Chernomor O, Schrempf D, Woodhams MD, von Haeseler A, Lanfear R (2020). IQ-TREE 2: new models and efficient methods for phylogenetic inference in the genomic era. Mol Biol Evol.

[59] Minh BQ, Hahn MW, Lanfear R (2020). New methods to calculate concordance factors for phylogenomic datasets. Mol Biol Evol.

[60] Chifman J, Kubatko L (2014). Quartet inference from SNP data under the coalescent model. Bioinformatics.

[61] Swofford DL: PAUP*. Phylogenetic Analysis Using Parsimony (*and Other Methods). Version 4.0b10, vol. Version 4.0. Sunderland, Massachusetts: Sinauer Associates; 2002.

[62] Pickrell JK, Pritchard JK (2012). Inference of population splits and mixtures from genome-wide allele frequency data. PLoS Genet.

[63] Humphries P (1995). Life history, food and habitat of southern pygmy perch, *Nannoperca australis*, in the Macquarie River. Tasmania Mar Freshw Res.

[64] Excoffier L, Dupanloup I, Huerta-Sanchez E, Sousa VC, Foll M (2013). Robust demographic inference from genomic and SNP data. PLoS Genet.

[65] Xue AT, Hickerson MJ (2017). multi-dice: r package for comparative population genomic inference under hierarchical co-demographic models of independent single-population size changes. Mol Ecol Resour.

[66] Csilléry K, François O, Blum MGB (2012). abc: an R package for approximate Bayesian computation (ABC). Methods Ecol Evol.

[67] Thuiller W, Lafourcade B, Engler R, Araújo MB (2009). BIOMOD—a platform for ensemble forecasting of species distributions. Ecography.

[68] Brown JL, Hill DJ, Dolan AM, Carnaval AC, Haywood AM (2018). PaleoClim, high spatial resolution paleoclimate surfaces for global land areas. Sci Data.

[69] Barbet-Massin M, Jiguet F, Albert CH, Thuiller W (2012). Selecting pseudo-absences for species distribution models: how, where and how many?. Methods Ecol Evol.

[70] Hao T, Elith J, Guillera-Arroita G, Lahoz-Monfort JJ (2019). A review of evidence about use and performance of species distribution modelling ensembles like BIOMOD. Divers Distrib.

[71] Waters JM, Burridge CP, Craw D (2019). The lasting biological signature of Pliocene tectonics: reviewing the re-routing of Australia’s largest river drainage system. J Biogeogr.

[72] Liu L, Xi Z, Davis CC (2015). Coalescent methods are robust to the simultaneous effects of long branches and incomplete lineage sorting. Mol Biol Evol.

[73] Byrne M (2008). Evidence for multiple refugia at different time scales during Pleistocene climatic oscillations in southern Australia inferred from phylogeography. Quat Sci Rev.

[74] Hesse PP, Magee JW, van der Kaars S (2004). Late Quaternary climates of the Australian arid zone: a review. Quat Int.

[75] Fitzsimmons KE, Cohen TJ, Hesse PP, Jansen J, Nanson GC, May J-H, Barrows TT, Haberlah D, Hilgers A, Kelly T (2013). Late Quaternary palaeoenvironmental change in the Australian drylands. Quat Sci Rev.

[76] Aitken SN, Yeaman S, Holliday JA, Wang T, Curtis-McLane S (2008). Adaptation, migration or extirpation: climate change outcomes for tree populations. Evol Appl.

[77] Woodward GMA, Malone B (2002). Patterns of abundance and habitat use by *Nannoperca obscura* (Yarra pygmy perch) and *Nannoperca australis* (southern pygmy perch). Proc R Soc Vic.

[78] Prentis PJ, Wilson JR, Dormontt EE, Richardson DM, Lowe AJ (2008). Adaptive evolution in invasive species. Trends Plant Sci.

[79] Williams JL, Hufbauer RA, Miller TEX (2019). How evolution modifies the variability of range expansion. Trends Ecol Evol.

[80] Bridle JR, Vines TH (2007). Limits to evolution at range margins: when and why does adaptation fail?. Trends Ecol Evol.

[81] Bouzat JL (2010). Conservation genetics of population bottlenecks: the role of chance, selection, and history. Conserv Genet.

[82] Szűcs M, Vahsen ML, Melbourne BA, Hoover C, Weiss-Lehman C, Hufbauer RA (2017). Rapid adaptive evolution in novel environments acts as an architect of population range expansion. PNAS.

[83] Pavlova A, Beheregaray LB, Coleman R, Gilligan D, Harrisson KA, Ingram BA, Kearns J, Lamb AM, Lintermans M, Lyon J (2017). Severe consequences of habitat fragmentation on genetic diversity of an endangered Australian freshwater fish: a call for assisted gene flow. Evol Appl.

